# Clinical phenotypes and prognoses of microscopic polyangiitis based on kidney biopsies

**DOI:** 10.1186/s13075-023-03218-0

**Published:** 2023-12-07

**Authors:** Juan Wang, Rui Li, Wenyan Zhou, Yanwei Lin, Xiaodong Wang, Shuang Ye, Liangjing Lu, Minfang Zhang, Sheng Chen

**Affiliations:** 1 Department of Rheumatology, Ren Ji Hospital, Shanghai Jiao Tong University School of Medicine, Shanghai, China; 2 Renal Division, Ren Ji Hospital, Shanghai Jiao Tong University School of Medicine, Shanghai, China

**Keywords:** Microscopic polyangiitis, Kidney biopsy, Clinical phenotypes, Prognosis

## Abstract

**Background:**

To classify the different clinical phenotypes and compare the distinct prognoses of microscopic polyangiitis (MPA).

**Methods:**

A retrospective analysis of 436 patients with anti-neutrophil cytoplasmic antibody (ANCA)-associated vasculitides (AAV) from 2015 to 2022 was conducted in our center, of which 90 patients were diagnosed with MPA and underwent renal biopsy.

**Results:**

Among the 90 MPA patients, 63% were female, and the median age at onset was 63 years (25th–75th percentile: 58–68). The median follow-up time was 26 months (25th–75th percentile: 10–53). We identified four subtypes: renal impairment type (cluster 1, 39%), pure type (cluster 2, 22%), systemic inflammation type (cluster 3, 26%), and rapid progress type (cluster 4, 13%). Cluster 1, characterized by renal dysfunction at onset (80%), demonstrated poor prognoses with only 26% achieved complete remission (CR), 11% dying, and 19% developing renal failure. In contrast, patients in cluster 2, exclusively female, most had only kidney involvement showed the best prognoses with 55% achieving CR and none experiencing death or renal failure within 10 years. Cluster 3 mostly consisted of males; high fever and C-reactive protein levels were the primary characteristics. These cases exhibited moderate prognoses with 53% achieving CR, 9% dying, and 4% developing renal failure. Finally, patients in cluster 4, which was characterized by rapidly progressive glomerulonephritis, had the worst prognoses, with none achieving CR, 8% dying, and 75% developing renal failure despite aggressive treatment.

**Conclusions:**

MPA is classified into four subtypes with distinct clinical manifestations and prognoses.

**Supplementary Information:**

The online version contains supplementary material available at 10.1186/s13075-023-03218-0.

## Background

Microscopic polyangiitis (MPA) is a type of necrotizing vasculitis in small vessels and is part of a family of diseases known as anti-neutrophil cytoplasmic antibody (ANCA)-associated vasculitides (AAV). MPA is frequently associated with fever, arthralgia/myalgia, purpuric skin lesions, and mononeuritis multiplex without granulomatous disease [1]. The kidneys and lungs are the most frequently reported organs affected in MPA, with almost 100% of patients having kidney involvement, which is typically asymptomatic until advanced renal failure occurs [2]. As a result, it is essential to detect microhematuria, erythrocyte casts, and non-nephrotic proteinuria in the urine of patients with renal involvement before the creatinine level increases. Missing or delaying a diagnosis of renal involvement could have potentially fatal consequences, since renal function determines survival and the likelihood of end-stage renal disease (ESRD). A combination of estimated renal function at presentation and histopathological parameters has been found to be the most effective method for predicting renal outcomes [3]. Berden et al. [4] defined four subclasses based on a histopathological classification: focal, crescentic, mixed, and sclerotic, with the best kidney outcomes found in the focal group and the worst outcomes in the sclerotic group. Some studies have shown similar outcomes for the crescentic and mixed groups, while others have found that mixed groups perform significantly better [4, 5].

While the clinical and pathological characteristics of granulomatosis with polyangiitis (GPA) and MPA were considered to be part of a single disease spectrum for several years, there is growing evidence that genetic associations, epigenetic control of major histocompatibility complex, and antigen function support the categorization of AAV by ANCA specificity (proteinase 3(PR3)-ANCA or myeloperoxidase (MPO)-ANCA) [6]. Disease clinical manifestations, histopathology, relapses, and outcomes have also been differentiated by means of this serological classification [3, 7]. The clinical presentation of MPA and the absence of anti-PR3 antibodies are associated with a reduced risk of relapse during follow-up. Moreover, marked geographic and ethnic differences have been observed for MPA and GPA, with MPA being more frequent than GPA in southern Europe [8] and almost exclusively presented in Japanese and Chinese patients [9, 10]. Despite this, there is currently no report on MPA subtypes.

Data mining techniques using clinical data have been reported as a promising strategy for understanding the complexity and heterogeneity of some rheumatic diseases and for determining therapeutic approaches and risk stratification. In this study, we describe our experience with 90 MPA patients who underwent kidney biopsies and were followed up at a single center. We aimed to define distinct clinical phenotypes for MPA.

### Patients and methods

A retrospective analysis of 436 patients with AAV from January 2015 to January 2022 was conducted in the Rheumatology Department at Shanghai Renji Hospital, of which 90 patients were diagnosed with MPA and underwent renal biopsy. Patients were included if they had sufficient clinical and laboratory data at diagnosis and during follow-up and if their kidney biopsy specimen was suitable for histopathologic classification. The study was approved by the ethics committee of Renji Hospital (IRB approval number: [2017] 201), and all patients consented to participate in the study. The patients were followed up until renal replacement therapy or death, or until December 2022. Of these, 84 (93%) patients completed follow-up, with a mean follow-up duration of 26 months (range 10–53 months). All patients met the diagnostic criteria for MPA set forth by the American College of Rheumatology [11] or the Chapel Hill Consensus Conference [12]. Patients with vasculitis secondary to drugs, infections, or systemic autoimmune diseases were excluded.

Clinical and laboratory data were obtained from the patients’ electronic records or the archived clinical charts at diagnosis, at month 6, and at last follow-up. The remission rate, relapse rate, infection rate, survival rate, chronic kidney disease (CKD) stage, and kidney failure were recorded. Kidney syndromes or abnormalities at presentation were defined as follows: rapid deterioration in kidney function (estimated glomerular filtration rate (eGFR) decline ≥ 50% in up to 3 months) for rapidly progressive glomerulonephritis (RPGN) [13], nephrotic-range proteinuria for urinary protein excretion ≥ 3.5 g/m^2^ per 24 h, and isolated urinary abnormalities including proteinuria or glomerular hematuria (more than three cells per high-power field, red cell casts, or ≥ 11 cells/ml on urinalysis) [14]. The eGFR was calculated using the modified Schwartz formula [15]. CKD was staged following the National Kidney Foundation Kidney Disease and Outcome Quality Initiative criteria. Kidney failure was defined as dialysis dependence (evaluated acutely and confirmed after 3 months) or transplantation. Kidney survival indicated the time from diagnosis to kidney failure or last follow-up. Kidney biopsy specimens were reassessed by pathologists and classified as focal, crescentic, sclerotic, or mixed according to Berden et al.’s classification [4]. Complete remission (CR) was defined as a Birmingham Vasculitis Activity Score (BVAS) of zero and an oral prednisolone dose of 10 mg/d or lower, partial remission (PR) was defined as persistence of minor proteinuria or hematuria, while relapse was defined as new emergence or recurrence of 1 or more items of the BVAS (version 3) after remission [16].

To identify possible subtypes of MPA, we performed a cluster analysis of all 90 patients based on clinical features and laboratory information. Variables such as sex, age, renal impairment as the first sign, dialysis treatment at the time of diagnosis, organ involvement, fever, arthritis, myalgia, weight loss, rash, MPO, BVAS onset, C-reactive protein, albumin, serum creatinine, eGFR, 24-h proteinuria, and renal biopsy pathological patterns were used in the analysis. In order to identify groups in data that a combination of numeric and categorical variables, traditional and classic clustering algorithms such as K-means are no longer available. So, we used the k-prototypes clustering method to fit the model, which was designed for clustering mixed-type data, by the “clustMixType” package (version 0.3–9) [17] in the R software (version 4.2.2). We conducted hypothesis testing to identify variables that significantly differed between the clusters. To evaluate the correlation between our classification results and the prognosis of patients with MPA, we examined the renal outcomes and overall survival rates of patients who completed follow-up within each cluster.

### Statistical analysis

Continuous variables were compared with one-way ANOVA test. Categorical variables were compared using the Fisher’s exact test. Survival data were analyzed via Kaplan–Meier plots and compared with the log-rank (Mantel-Cox) test. Statistical results were generated with Prism 9 (9.5.0) software. *P* < 0.05 (two tail) was considered significant.

## Results

### Patient characteristics

Our center enrolled a total of 436 patients with AAV between 2015 and 2022, out of which 90 patients were diagnosed with MPA and underwent renal biopsy. These patients had adequate clinical and laboratory data available at the time of diagnosis and during their follow-up period. The main characteristics of the patients are presented in Table 1. The majority of patients were female (63%) and the median (interquartile range, IQR) age at diagnosis was 63 (58–68) years. The most common symptoms were fever and arthralgia. Kidneys and lungs were the organs most frequently affected. Renal involvement was usually severe in MPA, with a mean eGFR of 25 (12–50) ml/min per 1.73 m^2^, and renal impairment was the most common kidney syndrome, observed in 44 (49%) patients. Thirty (33%) patients had RPGN, and 12 (13%) patients required kidney replacement therapy (KRT) at onset. Eleven patients (12%) were classified as focal, 31 (35%) as mixed-class, 28 (31%) as crescentic, and 20 (22%) as sclerosing. In this cohort, 61 (68%) patients had pulmonary involvement, with usual interstitial pneumonia (UIP) or nonspecific interstitial pneumonia (NSIP) observed in 42 (47%) patients. Sixteen (18%) cases had bronchiectasis, 2 (2%) patients had alveolar hemorrhage, and 1 (1%) case had a pulmonary mass.

**Table 1:** Clinical characteristics of 90 patients at baseline

	All	Renal impairment type	Pure type	Systemic inflammation type	Rapid progress type	*P*
Clinical characteristics						
Number, *n* (%)	90 (100)	35 (39)	20 (22)	23 (26)	12 (13)	
Female, *n* (%)	57 (63)	25 (71)	18 (90)	7 (30)	7 (58)	0.0004^*^
Age, median (25th–75th)	63 (58–68)	63 (58–69)	59 (45–66)	65 (61–67)	63 (60–68)	0.0933
Symptoms to diagnosis interval, months, median (25th–75th)	2 (1–6)	2 (1–12)	5 (1–12)	1 (1–3)	3 (1–4)	0.1194
Symptoms, *n* (%)						
Fever	25 (28)	1 (3)	2 (10)	20 (87)	2 (17)	< 0.0001^*^
Weight loss	3 (3)	2 (6)	0 (0)	0 (0)	1 (8)	0.3871
Rash	1 (1)	1 (3)	0 (0)	0 (0)	0 (0)	0.6619
Arthritis	9 (10)	3 (9)	4 (20)	2 (9)	0 (0)	0.2984
Myalgia	4 (4)	0 (0)	1 (5)	3 (13)	0 (0)	0.102
Renal impairment at the onset	44 (49)	28 (80)	4 (20)	5 (22)	7 (58)	< 0.0001^*^
Repaid progression	30 (33)	10 (29)	2 (10)	7 (30)	11 (92)	< 0.0001^*^
Dialysis at diagnosis	12 (13)	1 (3)	0 (0)	1 (4)	10 (83)	< 0.0001^*^
Cardiovascular symptoms	6 (7)	4 (11)	0 (0)	0 (0)	2 (17)	0.0989
Digestive symptoms	1 (1)	1 (3)	0 (0)	0 (0)	0 (0)	0.6619
Nervous symptoms	4 (4)	1 (3)	0 (0)	3 (13)	0 (0)	0.1271
ENT involvement	5 (6)	2 (6)	0 (0)	3 (13)	0 (0)	0.2268
Renal biopsy, *n* (%)	90 (100)					
Focal	11 (12)	3 (9)	3 (15)	5 (22)	0 (0)	0.2415
Mixed	31 (34)	7 (20)	13 (65)	11 (48)	0 (0)	0.0002^*^
Crescent	28 (31)	17 (49)	1 (5)	3 (13)	7 (58)	0.0003^*^
Sclerotic	20 (22)	8 (23)	3 (15)	4 (17)	5 (42)	0.3147
Pulmonary involvement, *n* (%)	61 (67)	24 (69)	3 (15)	23 (100)	11 (92)	
Bronchiectasis	16 (18)	4 (11)	1 (5)	9 (39)	2 (17)	0.0156^*^
UIP or NSIP	42 (47)	19 (54)	2 (10)	14 (61)	7 (58)	0.0027^*^
Alveolar hemorrhage	2 (2)	0 (0)	0 (0)	0 (0)	2 (17)	0.004^*^
Laboratory results, median (25th–75th)						
MPO-ANCA titers	5 (3–6)	5 (2–6)	4 (3–5)	5 (4–7)	4 (3–7)	0.1734
BVAS	16 (12–18)	16 (14–16)	12 (10–13)	18 (16–19)	16 (13–20)	< 0.0001^*^
Cr, μM	197 (121–349)	243 (184–308)	103 (82–163)	127 (109–277)	588 (490–966)	< 0.0001^*^
eGFR	25 (12–50)	21 (15–26)	54 (29–77)	50 (16–61)	7 (5–9)	< 0.0001^*^
Albumin, g/L	34 (30–36)	33 (30–38)	35 (33–38)	29 (27–35)	35 (29–36)	0.0364^*^
CRP, mg/L	18 (5–45)	21 (9–36)	3 (1–18)	102 (17–130)	12 (4–35)	< 0.0001^*^
24 h urine protein, g/L	1.5 (0.8–2.7)	1.5 (0.9–2.4)	1.9 (0.8–3.2)	1.0 (0.8–1.5)	3.1 (2.6–3.5)	0.0008^*^

*ENT *ear, nose, and throat, *UIP *usual interstitial pneumonia, *NSIP *nonspecific interstitial pneumonia, *MPO-ANCA * myeloperoxidase-anti-neutrophil cytoplasmic antibody, *BVAS *Birmingham Vasculitis Activity Score, *Cr *serum creatinine, *eGFR *estimated glomerular filtration rate, *CRP *C-reactive protein

^*^P < 0.01

### Treatment regimen and main outcome

All 90 consecutive patients were given remission-induction therapies. The majority (88%) received intravenous cyclophosphamide (IVC) pulses and steroids, while rituximab plus steroids was given to 6% of patients, and 1% received glucocorticoids alone. The remaining patients received various combinations of glucocorticoids, mycophenolate mofetil, methotrexate, and *Tripterygium wilfordii*. Four patients (4.4%) with severe renal involvement or diffuse alveolar hemorrhage received additional therapy with plasma exchange. Maintenance treatment in 92% of patients included azathioprine, methotrexate, mycophenolate mofetil, or low-dose cyclophosphamide (once every 3 months).

After 6 months, 79 patients (88%) were followed-up. Of these, 33% achieved CR, 33% achieved PR, 17% had uncontrolled disease progression, and 4.4% showed no response. In the final follow-up of 78 patients, 36% achieved CR, 20% achieved PR, 29% had uncontrolled disease progression, and 2.2% showed no response. During follow-up, 19% of patients experienced at least one recurrence, with a mean time to relapse of 24 (17–54.5) months. The main cause of relapse was discontinuation or withdrawal of immunosuppressive agents after infection.

The median (IQR) time to kidney failure or last follow-up was 22.5 (3.8–54.3) months. At diagnosis, 13% of patients were on KRT, while 11% were classified as kidney failure (CKD 5) at month 6 and 22% at last follow-up. Seventeen patients (19%) were on dialysis finally. Infection was a common occurrence during treatment, with 36% of patients developing an infection. The most common type was pulmonary infection (32%, *n* = 29), followed by herpes zoster (5.6%, *n* = 5). Seven patients (7.8%) ultimately died during the follow-up period. The causes of death were severe pulmonary infection in three patients, cerebral hemorrhage in one, sudden death in one, status epilepticus in one, and lung cancer in one. The 3-year and 5-year survival rates after diagnosis were 91.7% and 88.7%, respectively (Fig. 4).

### Clustering analysis

**Fig. 1:**
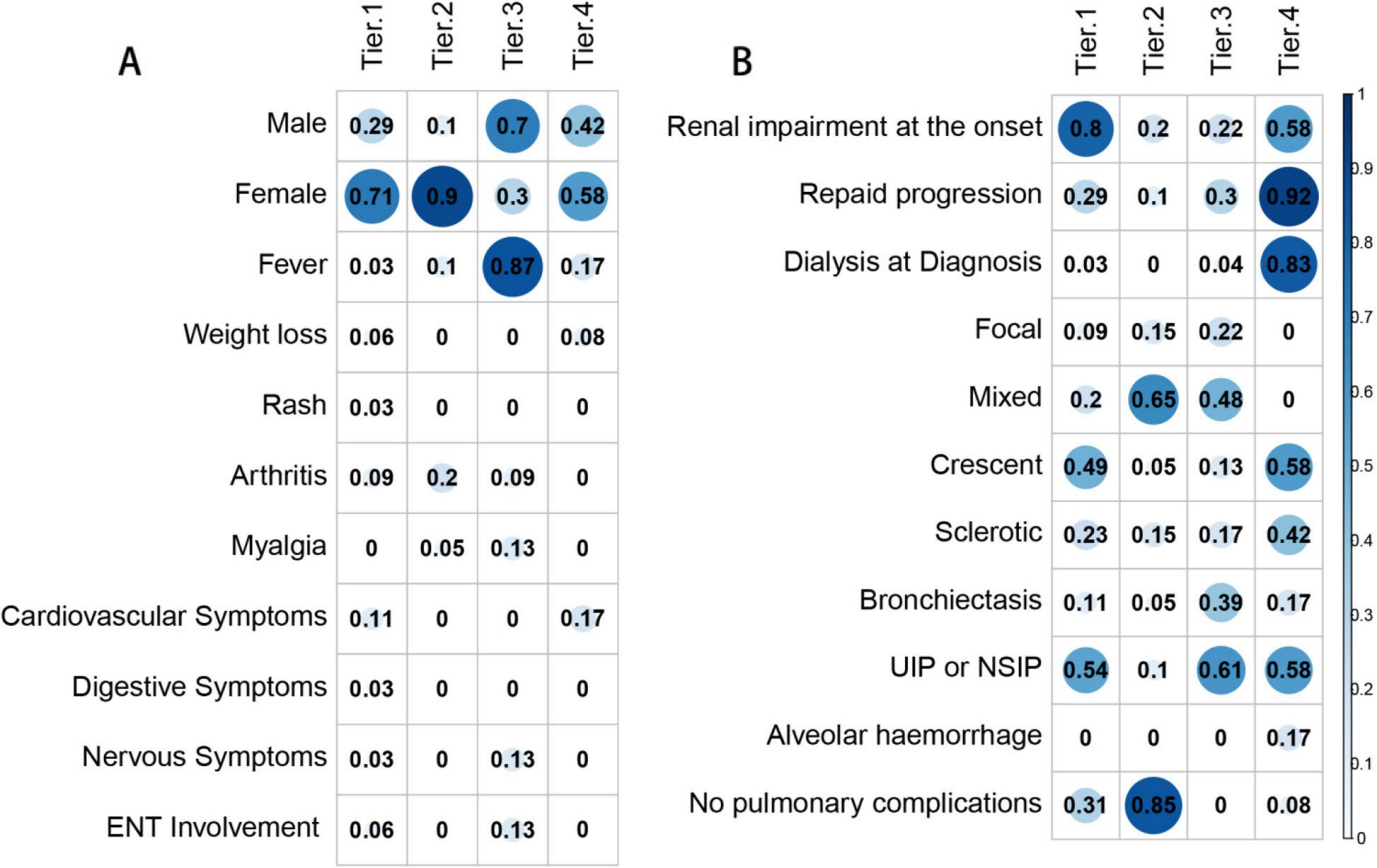
The correlations between clinical features and clusters. The correlation matrix indicating the percentage of a given feature (row) in a cluster (column). The size of each blue dot on the visualization indicating the proportion of patients in that cluster. *ENT*, ear, nose, and throat, *UIP*, usual interstitial pneumonia, *NSIP*, nonspecific interstitial pneumonia

**Fig. 2:**
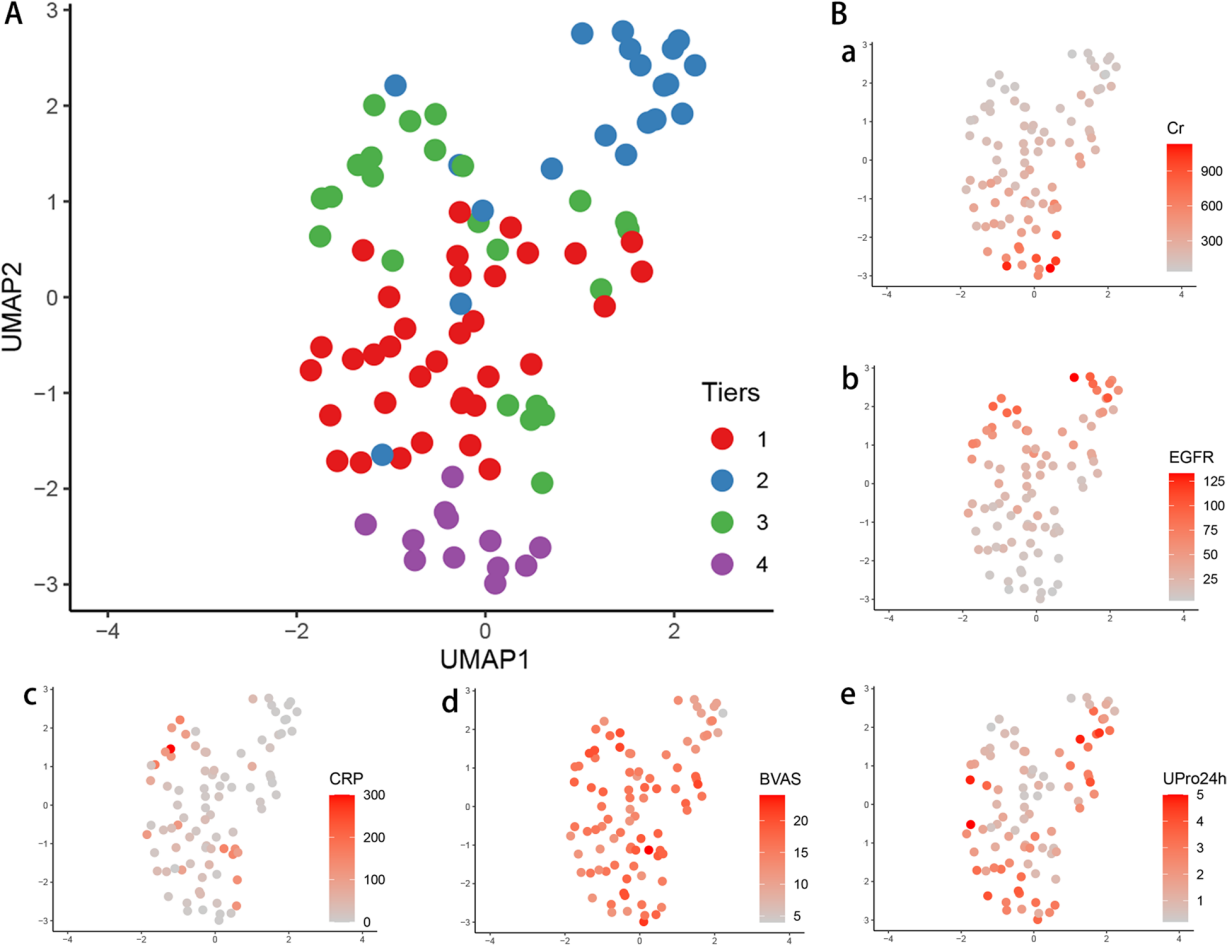
The identification of clinical subtypes of MPA patients. **A** A UMAP visualization of both clinical and laboratory data from 90 patients at baseline was performed, which revealed distinct grouping of patients into 4 clusters. **B** Laboratory results projected onto UMAP plots, including Cr (creatinine, a), eGFR (estimated glomerular filtration rate, b), CRP (C-reactive protein CRP, c), BVAS (Birmingham Vasculitis Activity Score, d), and total UPro24h (urine protein of 24 h, e). The scale showed the original data without transformation

**Fig. 3:**
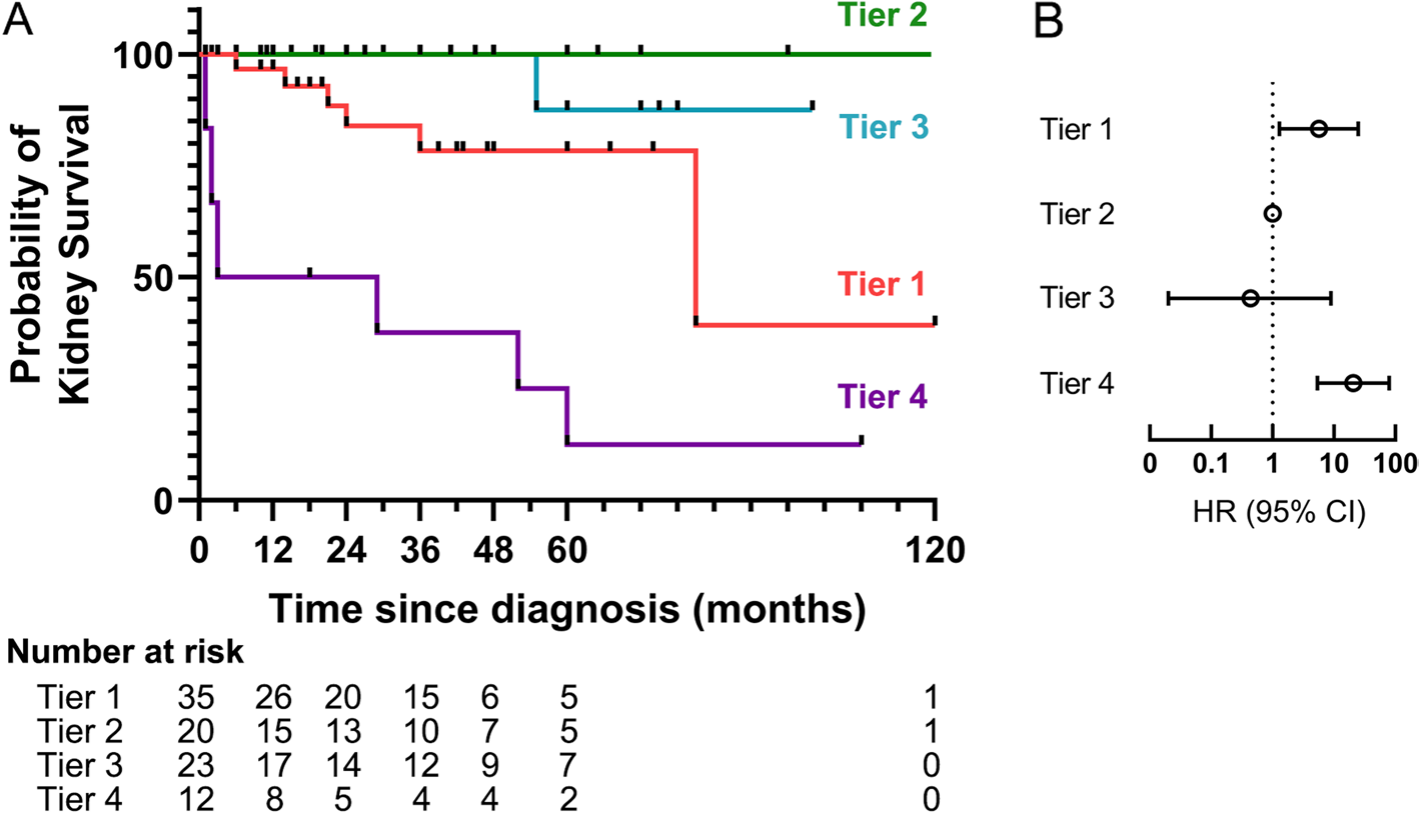
Kaplan–Meier estimates and forest plots indicating the risks of kidney survival, according to clusters

**Fig. 4:**
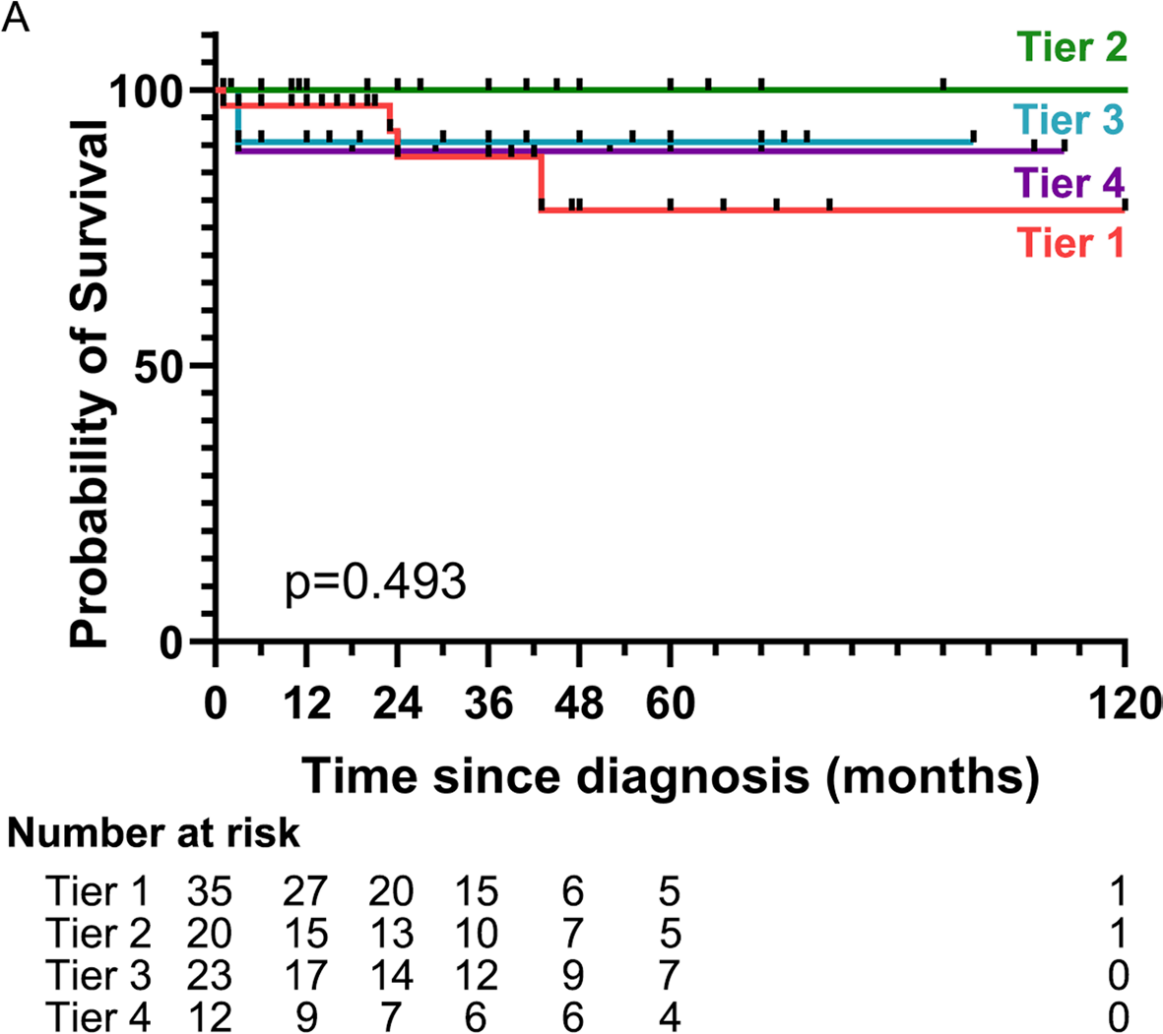
Kaplan–Meier estimates and forest plots indicating the risks of survival, according to clusters

In order to identify possible subtypes of MPA, we utilized a method of unsupervised clustering of the clinical characteristics. After fitting candidate cluster models using the k-prototypes clustering method, we concluded that the four-subgroup model had the most favorable fit statistics (Supplementary Fig. 1). These four subgroups were found to be clinically significant (Table 1, Figs. 1 and 2). Specifically, we defined the four groups as follows: renal impairment type (cluster 1), pure type (cluster 2), systemic inflammation type (cluster 3), and rapid progress type (cluster 4). Furthermore, we discovered that these subgroups were associated with different prognoses. We conducted an analysis of renal outcomes and survival among the four subgroups using Kaplan–Meier survival curves (Figs. 3 and 4). The distribution of the different CKD stages at baseline and last follow-up between the four subgroups were shown in Supplementary Fig. 2

As shown in Table 1 and Fig. 1, 35 patients (39%) were classified as having the renal impairment type (cluster 1). Among these patients, 71.4% were female, and 80% exhibited renal impairment and had a higher average concentration of serum creatinine (243 (184–308) μmol/L) at onset. About half of these patients had the crescentic types, and 69% experienced respiratory system involvement. The response to treatment was poor; only 26% achieved complete remission (CR), 43% of patients suffered from infection, 11% dying, and 17% developed renal failure at the final follow-up (Figs. 3 and 4, Supplementary Fig. 2).

In contrast, 20 patients (22%) were classified as cluster 2, almost all of whom were female (90%). Most of these patients had kidney involvement exclusively, and the histological types were mixed (65%), with crescentic (15%) and focal (15%) types. These patients exhibited the lowest levels of BVAS (12 (10–13)) and serum creatinine concentration (103 (82–163) μmol/L). The response to treatment was favorable; 55% received CR, and none of them died or progressed to renal failure. The total infection rate was only 10% during follow-up.

Patients in cluster 3 (26%, *n* = 23) were predominantly male (70%) and presented with fever and high C-reactive protein (CRP) levels as the main characteristics. Both the kidney and respiratory systems were all involved (100%), as well as ear, nose, and throat (ENT) (13%) and nervous system (13%). Nearly half of the histological types were mixed (48%). Despite with the highest BVAS levels (18 (16–19)), the creatinine level (127 (109–277) μmol/L) was relatively lower. The patients responded well to treatment, with 53% achieving CR, 9% dying, and 4% developing renal failure (Figs. 3 and 4, Supplementary Fig. 2).

During follow-up, we found that patients in this group had the highest infection rate (52%, *n* = 12).

Finally, cluster 4 (13%, *n* = 12) had the lowest proportion of patients and was characterized by rapidly progressive glomerulonephritis, with most patients requiring kidney replacement therapy (KRT) at the time of diagnosis. Some of these patients also exhibited diffuse alveolar hemorrhage (17%). Histological types showed two types: crescentic (58.3%) and sclerotic (41.7%). Despite aggressive treatment, none of the patients achieved CR, 8% dying, and almost all of them (75%) developed kidney failure and required dialysis (Figs. 3 and 4, Supplementary Fig. 2).

## Discussion

In this study, we aimed to analyze the clinical presentation and kidney outcome of Chinese patients with MPA, a common presentation of AAV. To the best of our knowledge, no previous reports have classified MPA subtypes based on clinical features and renal pathology. Our analysis of patients with biopsy specimen–proven ANCA-associated vasculitis led us to identify four distinct subtypes of MPA, namely renal impairment type, pure type, systemic inflammation type, and rapid progress type, each with different clinical manifestations and prognoses.

Our cohort comprised 57 (63%) female patients, with a median onset age of 63 (58–68) years [median (25th–75th percentile)]. This is consistent with previous studies showing an increased incidence of AAV with age, peaking between 60 and 70 years [18]. Although several studies have shown a slight male predominance in AAV [19, 20], we found a female predominance for MPA, as previously reported [21]. MPA is a multisystemic disease, with renal symptoms being common, but the disease is also associated with general symptoms such as fever, arthralgia/myalgia, purpuric skin lesions, and mononeuritis multiplex. Unlike GPA, ENT abnormalities are not typical for MPA. We observed that fever and arthralgia were the most frequent symptoms, with 100% of patients having kidney involvement and 61 (67%) cases having lung involvement. Our patients had a remarkably lower frequency of peripheral neuropathy (4%) than reported in other studies, which may be due to the fact that the kidney was the main affected organ in our cohort.

In line with current recommendations, remission-induction therapy for new onset organ-threatening or life-threatening AAV involves treatment with glucocorticoids in combination with either cyclophosphamide or rituximab [22, 23]. While there is no established remission rate for AAV, previous clinical studies have reported a remission rate of 70–90% [24, 25]. In our cohort, moderate/high doses of corticosteroids and IVC pulses or rituximab combinations were used to induce remission, which was achieved in 66% of cases at 6 months and 56% at the last follow-up, slightly lower than rates reported in previous clinical trials. This may be due to several factors, such as the more frequent and advanced kidney disease observed in MPA patients, as evidenced by the mean eGFR of 25 (12–50) ml/min per 1.73 m^2^ in our cohort [26, 27]. In addition, MPO-ANCA with features of irreversible kidney injury and poor response to immunosuppression [18], as well as lost follow-up patients in retrospective studies, may have led to an underestimation of the response rate.

The remission rate varied among the four types of patients, with eGFR or serum creatinine at baseline and kidney histology proving to be strong prognostic indicators of long-term kidney function. Renal remission was also dependent on renal function at baseline. In our cohort, patients with mixed biopsy specimens had a significantly better remission rate than those with crescentic biopsy specimens. Patients in cluster 2 and 3 had relatively lower creatinine levels at baseline, and half of the histological types were mixed, resulting in a good remission rate of 55% and 53%, respectively, at the last follow-up. However, in cluster 1, serum creatinine levels at baseline were high, and more than half of the histological types were crescentic, resulting in only 26% of patients achieving CR. In cluster 4, most patients required KRT at diagnosis, and there was no remission. Furthermore, 83% of cases had disease progression, and 75% developed renal failure in cluster 4. Kidney involvement was present in all patients, and renal impairment was the most common kidney syndrome, with a substantial number of patients presenting with RPGN in one-third of cases. Despite aggressive treatment, traditional immunosuppressive therapies only switched off systemic manifestations and were difficult to reverse the renal function during the pathogenesis of AAV. The occurrence of CKD followed a pattern similar to that of kidney survival, with most patients who reached CKD 3–5 doing so during the follow-up in our cohort. Therefore, for patients with poor baseline renal function, a relatively conservative treatment might be adopted.

Interestingly, we have found a unique subgroup (cluster 3) consisting predominantly of male patients with fever, high levels of CRP and BVAS, and a higher prevalence of bronchiectasis. Although presenting with prominent systemic inflammation, the renal prognoses and therapy response were moderate. Notably, this cluster had the highest probability of respiratory infection, highlighting the importance of infection prevention measures in managing these patients. What is more, the four types of patients had different rates of secondary infection. Patients with systemic inflammation had the highest probability of infection, followed by patients with the renal impairment type. The infection rates were relatively low at 10% and 25% in clusters 2 and 4, respectively. Regarding relapse, 17 (19%) cases experienced at least one recurrence of the disease, and there were no statistically significant differences among the four clusters.

Several limitations should be considered when interpreting our study. First, the retrospective design may have introduced recall and selection errors. Second, since only MPA patients undergoing renal biopsy were included, our findings may not be generalizable to all MPA patients. Third, the sample size was small and further studies are needed to validate our observations.

## Conclusions

In this study, we report on a recent series of 90 patients with MPA proven by kidney biopsy specimens from a single center. We identified four subtypes with distinct clinical manifestations and prognoses.

### Supplementary Information

**Additional file 1:** **Supplementary Fig. 1.** The scree plot used for choosing an appropriate number of clusters for our data set. The objective function (*E*) is given by multiple times of calculation for varying numbers of clusters, and the number of clusters is chosen as the minimum *k* from whereon no strong improvements of *E* are possible. In this figure, a relatively good elbow is visible at clusters equal 4.

**Additional file 2: Supplementary Fig. 2.** CKD stages at baseline (left) and last follow-up (right) in the different clusters.

## Data Availability

There are no new data associated with this article.

## Abbreviations

ANCA: Anti-neutrophil cytoplasmic antibody
AAV: Anti-neutrophil cytoplasmic antibody (ANCA)-associated vasculitides
BVAS: Birmingham Vasculitis Activity Score
CR: Complete remission
CKD: Chronic kidney disease
CRP: C-reactive protein
ESRD: End-stage renal disease
eGFR: Estimated glomerular filtration rate
ENT: Ear nose and throat
GPA: Granulomatosis with polyangiitis
PR3: Proteinase 3
MPA: Microscopic polyangiitis
MPO: Myeloperoxidase
RPGN: Rapidly progressive glomerulonephritis
PR: Partial remission
KRT: Kidney replacement therapy
UIP: Usual interstitial pneumonia
NSIP: Nonspecific interstitial pneumonia
IVC: Intravenous cyclophosphamide
IQR: Interquartile range
