# Supplementary figures and images for: Clinical phenotypes and prognoses of microscopic polyangiitis based on kidney biopsies
Source: Arthritis Res Ther. 2023 Dec 7;25:239. doi: 10.1186/s13075-023-03218-0 (PMC10702060; doi:10.1186/s13075-023-03218-0)

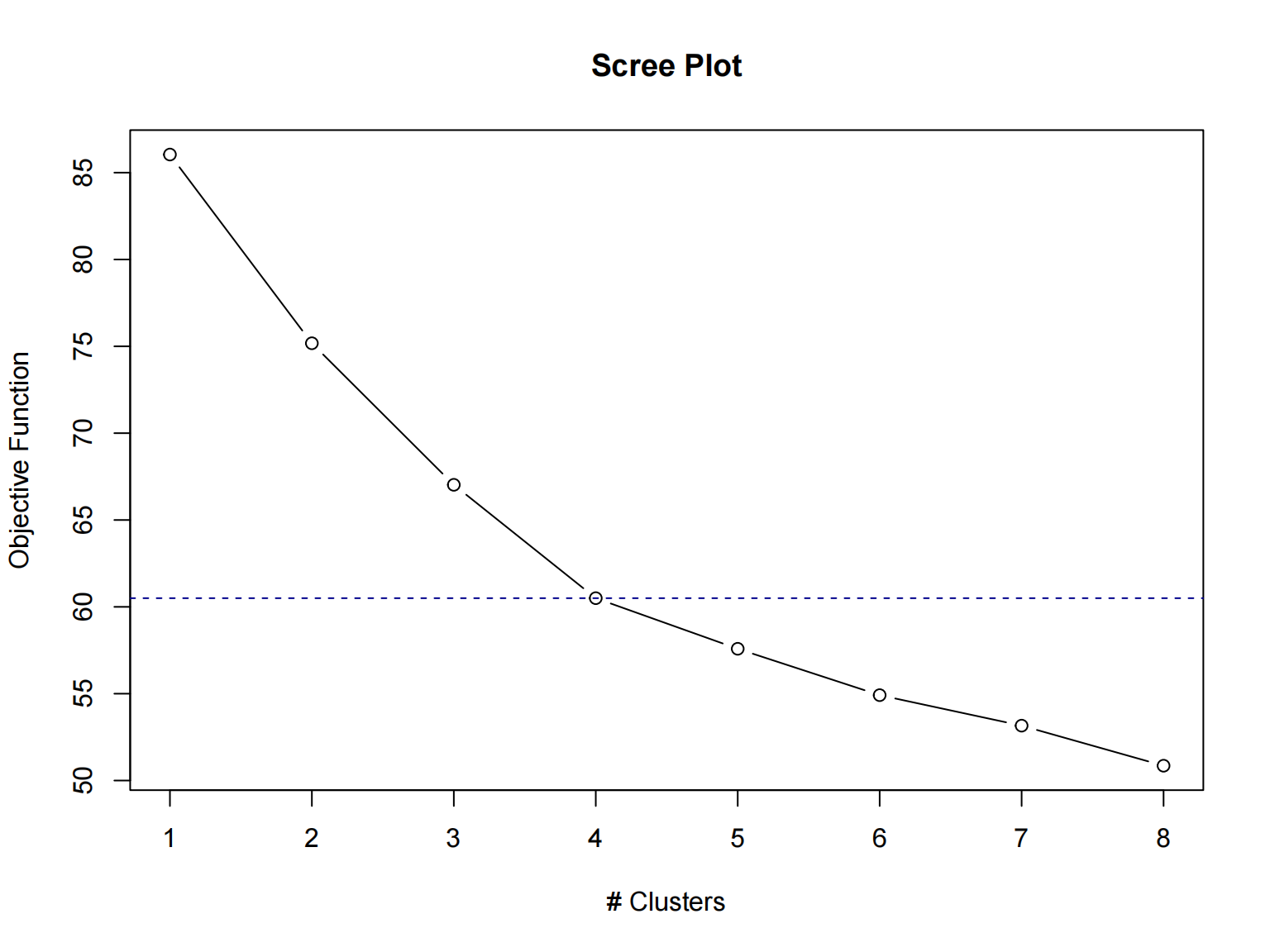

Supplement: Supplementary file 1 — Additional file 1: Supplementary Fig. 1. The scree plot used for choosing an appropriate number of clusters for our data set. The objective function (E) is given by multiple times of calculation for varying numbers of clusters, and the number of clusters is chosen as the minimum k from whereon no strong improvements of E are possible. In this figure, a relatively good elbow is visible at clusters equal 4. [file 13075_2023_3218_MOESM1_ESM.tif]

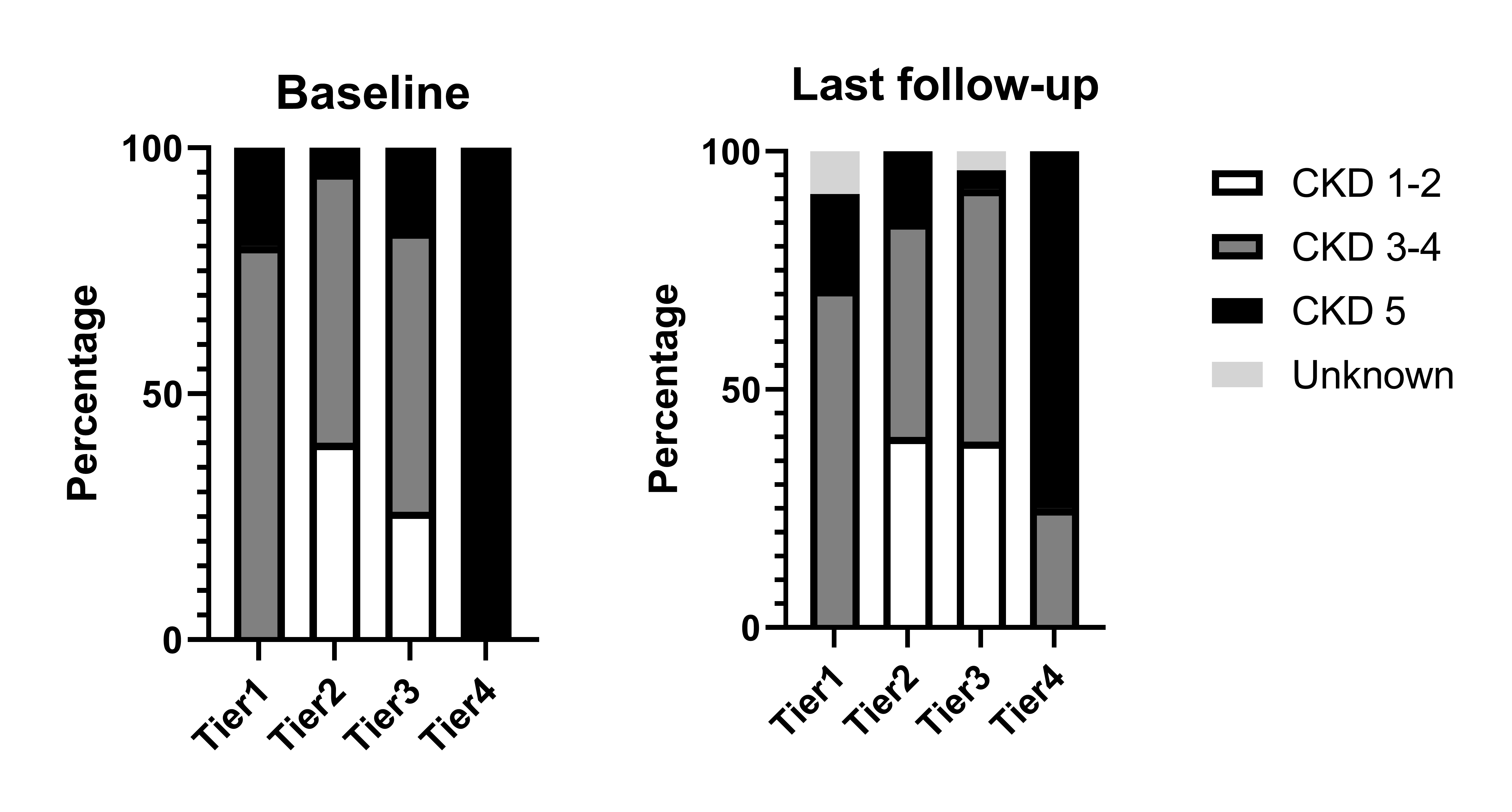

Supplement: Supplementary file 2 — Additional file 2: Supplementary Fig. 2. CKD stages at baseline (left) and last follow-up (right) in the different clusters. [file 13075_2023_3218_MOESM2_ESM.tif]
